# Genetic Factors Affecting Seasonality, Mood, and the Circadian Clock

**DOI:** 10.3389/fendo.2018.00481

**Published:** 2018-08-23

**Authors:** Corrado Garbazza, Francesco Benedetti

**Affiliations:** ^1^Centre for Chronobiology, University of Basel, Basel, Switzerland; ^2^Transfaculty Research Platform Molecular and Cognitive Neurosciences, University of Basel, Basel, Switzerland; ^3^Psychiatry and Clinical Psychobiology, Division of Neuroscience, Scientific Institute and University Vita-Salute San Raffaele, Milan, Italy

**Keywords:** seasonality, mood disorders, clock genes, circadian rhythm, seasonal affective disorder

## Abstract

In healthy humans, seasonality has been documented in psychological variables, chronotype, sleep, feeding, metabolic and autonomic function, thermoregulation, neurotransmission, and hormonal response to stimulation, thus representing a relevant factor to account for, especially when considering the individual susceptibility to disease. Mood is largely recognized as one of the central aspects of human behavior influenced by seasonal variations. This historical notion, already mentioned in ancient medical reports, has been recently confirmed by fMRI findings, which showed that seasonality in human cognitive brain functions may influence affective control with annual variations. Thus, seasonality plays a major role in mood disorders, affecting psychopathology, and representing the behavioral correlate of a heightened sensitivity to factors influencing circannual rhythms in patients. Although the genetic basis of seasonality and seasonal affective disorder (SAD) has not been established so far, there is growing evidence that factors affecting the biological clock, such as gene polymorphisms of the core clock machinery and seasonal changes of the light-dark cycle, exert a marked influence on the behavior of patients affected by mood disorders. Here we review recent findings about the effects of individual gene variants on seasonality, mood, and psychopathological characteristics.

## Introduction

Seasonality is a central aspect of environmental variability, which has strongly influenced life on Earth by driving the development of biodiversity among living organisms and the evolution of extreme physiological adaptations and behaviors, such as migration and hibernation. In most species, periodic variations of environmental conditions, particularly those related to the light-dark cycle and depending on latitude, season, and time of day, require that internal timing mechanisms induce the adaption of behavioral or physiological functions to such changes (1).

Biological rhythms with an approximate 24-h period, close to the daily light-dark cycle, are known as circadian rhythms and defined by three fundamental properties: persistence of an ~24-h rhythm, entrainability, and temperature compensation (2). The observation that these endogenous processes are also present among organisms such as cyanobacteria, which represent one of the earliest and most primitive species, suggests that circadian rhythms implicated a clear evolutionary advantage (1).

## Clock genes and mood regulation

**Figure 1:**
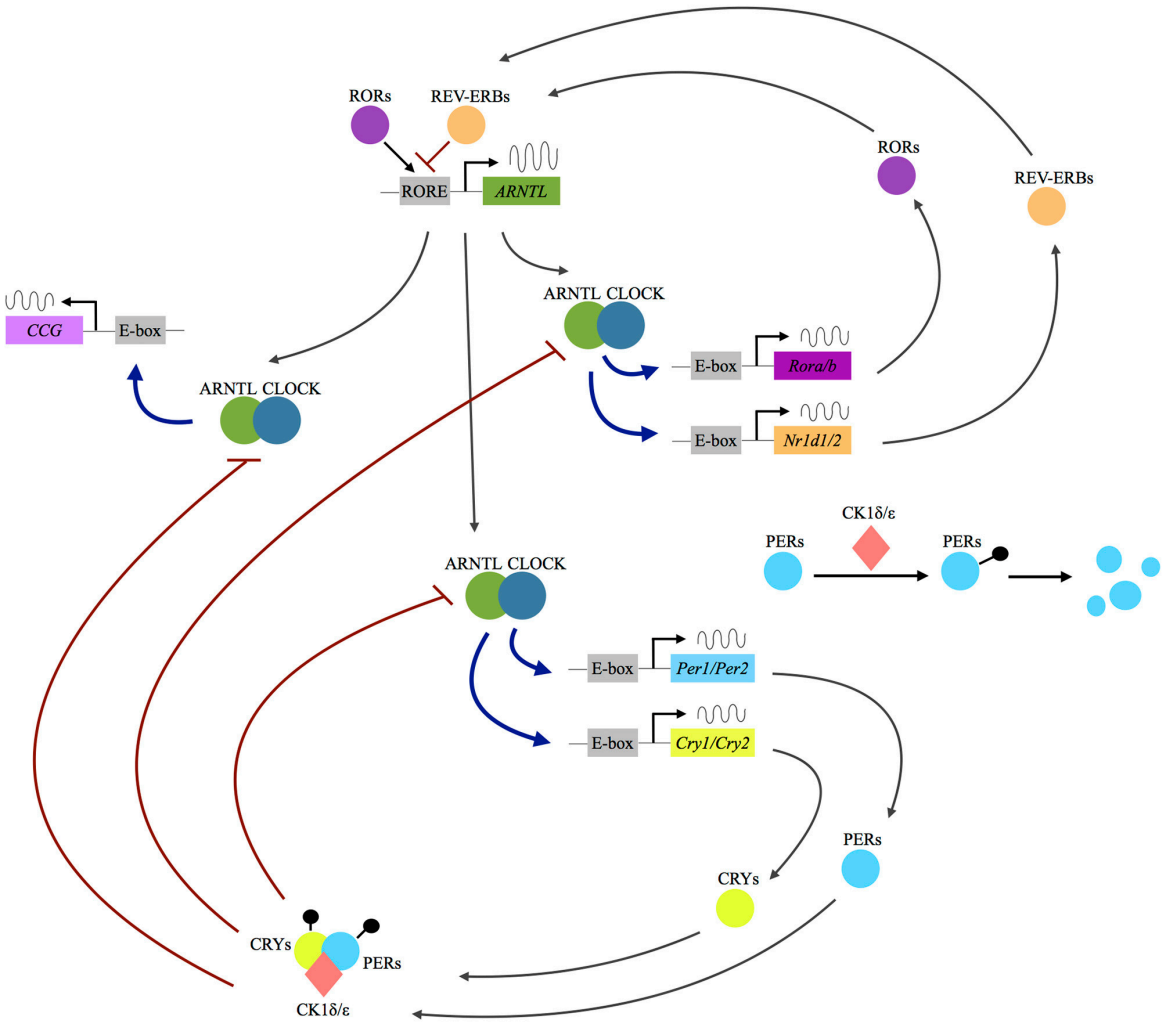
Molecular mechanisms of the circadian clockwork. Following the dimerization of the transcription factors circadian locomotor output cycles kaput (CLOCK) and brain and muscle Arnt-like (ARNTL) or neuronal pas domain protein 2 (NPAS2) proteins, the expression of the clock proteins period (PER1, PER2, PER3) and cryptochrome (CRY1, CRY2) is initiated. The PER and CRY proteins interact with the serine/threonine kinases casein kinase 1 δ/ε, (CK1 δ/ε) and form a complex allowing nuclear translocation. In the nucleus they act as inhibitors of CLOCK:ARNTL (or CLOCK:NPAS2) activity and therefore block their own expression. An additional feedback loop is generated by CLOCK:ARNTL (or CLOCK:NPAS2) mediated transcription of REV-ERB and rar-related orphan receptor A/B (RORA/B), which in turn also regulate ARNTL transcription. Up to 10% of the human genome is under the influence of the molecular clock (clock-controlled genes, CCG). RORE: ROR response element.

At the cellular level, circadian rhythms are generated by a core molecular clock consisting of multiple transcriptional/translational feedback loops (3). The transcription factors circadian locomotor output cycles kaput (CLOCK) and brain and muscle Arnt-like (ARNTL), or neuronal pas domain protein 2 (NPAS2) proteins, dimerize and initiate the expression of the clock proteins PERIOD (PER1, PER2, PER3), and CRYPTOCHROME (CRY1, CRY2). With rising accumulation, PER1-3 and CRY1/2 inhibit CLOCK:ARNTL (or CLOCK:NPAS2) activity and therefore block their own expression (3). An additional feedback loop is generated by CLOCK:ARNTL (or CLOCK:NPAS2) mediated transcription of REV-ERB and RORs, which in turn also regulate ARNTL transcription (see Figure 1).

As recently reviewed by Albrecht, there is already solid scientific evidence showing that the above-mentioned proteins “not only self-promote their own temporally fluctuating transcription, but also regulate the transcription of a large number of clock-controlled genes (CCGs) and/or modulate key molecular pathways via protein–protein interactions, such as the monoaminergic system, the HPA axis or neurogenic pathways” [(4), p. 1]. Several cellular processes in the brain are under the control of the circadian clock, including “differentiation, growth, motility and apoptosis, immune functions and neuroinflammation, neurogenesis, and neuroplasticity” [(5), p. 236]. A desynchronization of the circadian gene network and disruption of its downstream mechanisms has therefore widespread potential implications for a vast array of physiological processes.

Hampp et al. demonstrated that the functional triade of *PER2, ARNTL*, and *NPAS2* and their encoded proteins, directly regulate the activation of the monoamine oxidase A gene *(Maoa)*. In fact, the transcription and activity of the MAOA enzyme in the mesolimbic neurons is decreased in mice carrying a genetic deletion of the *Per2* gene, causing an increase of the dopamine levels and an altered neuronal activity in the striatum, as well as behavioral changes (6, 7).

Dopamine is an important neurotransmitter in the reward system, and its levels in the nucleus accumbens show a circadian rhythmicity (6, 8). Considering that many other brain areas of the reward system, including the ventral tegmental area, prefrontal cortex, and amygdala, are also involved in both mood regulation and clock genes expression, this suggests that the entire reward circuit may be under the influence of the circadian clock, via dopamine metabolism (5).

Cryptochromes (CRY2 and CRY1) are key components of the molecular clock, which drive several functions of the circadian pacemaker (9) and are necessary for the development of intercellular networks in the suprachiasmatic nucleus (10). CRY2 and CRY1 proteins are functionally repressors of the transcription-translation loops, and inhibitors of the cyclic adenosine monophosphate signal pathway (11–14). Due to these important molecular properties at the circadian clock level, it has been suggested that CRY2 and CRY1 may play a major role in the metabolism of glucose and lipids (15, 16) and contribute to mood regulation on daily basis, as well as to seasonal variations in mood and behavior (17).

Finally, *PER3* is one of the most robustly rhythmic genes in humans and animals, playing a significant role in the temporal organization of peripheral tissues and being associated with diurnal preference, mental disorders, non-visual responses to light, as well as brain and cognitive responses to sleep loss/circadian misalignment (18). Some genetic variants are supposed to interfere with the stabilizing effect of PER3 on PERIOD1/2 proteins, which play critical roles in circadian timing. These findings suggest that *PER3* may represent an important element of the missing molecular linkage between sleep and mood regulation by adapting these processes to seasonal changes (19).

## Clock genes in mood disorders

Several human population genetic studies have identified specific single nucleotide polymorphisms (SNPs) or variable number of tandem repeats (VNTR, see Supplementary Table 1) of different circadian clock genes that are associated with mood disorders (20, 21). These associations remain controversial, since most findings could either not be replicated or hold up to correction for multiple testing (22). From a pathophysiological point of view, recent experimental work, and mathematical models suggest that changes in period length and/or decreased amplitude of the circadian oscillation may depend on the impact of specific polymorphisms on the overall function in terms of structure and stability of a given clock protein (23). By as of yet poorly understood processes, the resulting functional changes of the clock-machinery and misalignment between clock-regulated functions and the environment can influence core psychopathological features of mood disorders, including the timing of onset and recurrence of illness episodes, individual symptomatology, and response to treatments (5).

### Depressive disorder

In depressive disorder (DD) (7), two *TIMELESS* polymorphisms have been found to be associated with excessive daytime fatigue among women, as well as a two-way interaction of *TIMELESS* and *ARNTL (rs1868049)* with early-morning awakening among men (24). Lavebratt et al. demonstrated that *RORA, PER2*, and *NPAS2* are associated with DD and the onset of depression within 3 years independently from financial strain (25). Both an increased or decreased *PER3* transcriptional activity may implicate a higher risk for MDD. In particular, Shi and colleagues identified a missense mutation in *hPER3 (hPER3-P856A)*, which slightly lengthens the circadian period and is related to MDD in females, by likely driving changes in clock-controlled genes as opposed to SCN timing. Moreover, the authors describe other sex-dependent associations of common polymorphisms with a *CLOCK* variant protective of MDD in males and *NPAS2* polymorphisms with association of MDD especially in females (26). *NPAS2* and *CRY1* were also linked with DD in a study by Soria et al. (27), with the latter finding replicated by Hua et al. (28), who, instead, did not find any association of *CRY2 (rs10838524)* with major depressive disorder (MDD). However, Kovanen et al. suggested that CRY2 and the protein kinase C delta binding protein (PRKCDBP, or CAVIN3) variants may represent risk factors for MDD (29). Finally, the best association between a SNP and MDD based on genome-wide association studies has been found for *NR1D1* (30).

### Bipolar disorder

The observation that patients with bipolar disorder (BD) show alterations in circadian rhythms, and recurrent fluctuations of mood and sleep disturbances (31) has suggested a possible dysfunction of the biological clock in the pathogenesis of BD (32). Moreover, since heritability in BD is estimated to be as high as 85% (33), an increasing interest in identifying genetic risk factors has supported different association studies looking at the link between BD and some core clock genes (7).

Significant SNPs associations with bipolar 1 disorder were found for *TIMELESS* and *ARNTL* (34), as well as for *NPAS2, RORB 9*, and *CRY2* (35). Gonzalez et al. performed a family-based association study of circadian genes and BD in a Latino population, reporting nominal associations between SNPs of *CSNK1E, ARNTL, CSNK1D, CLOCK*, as well as statistically significant associations between *CSNK1E* and *ARNTL* haplotypes and BD, with either increased susceptibility or protective effect against the development of the disorder respectively (36). Shi et al. demonstrated the three-way interaction of *BHLHE40, TMEM165 (transmembrane protein 165)*, and *CSNK1E* with bipolar disorder (37), while McGrath et al. focusing their analysis on the *RORA* and *RORB* genes, found that 4 *RORB* SNPs were associated with bipolar 1 disorder (38). Etain et al. indicated a significant association of *TIMELESS* and of *RORA* with BD (39), while Lee et al. found *CLOCK 3111T/C* to have significant allelic and genotypic associations with the disease (40). *GSK3beta* was associated with bipolar type 2 disorder in women (41). General associations of *NR1D1* (42) and of *VIP* (27) with BD were also reported. In genome-wide association studies, the associations of *ARNTL, GSK3beta, RORB*, and *CRY 2* gene variants with BD have gained further support (30, 43).

### Circadian genes and phenotypic characteristics in bipolar disorder

Genetic polymorphisms influencing clock genes functions have shown major effects on the phenotypic clinical features of disease (44). A SNP in *CLOCK* gene, which is known to influence diurnal preference in healthy subjects (45), also impacts on bipolar patients, leading to worsening of insomnia, higher evening activity and delayed sleep onset. Carriers of the allelic C variant also showed a higher episode recurrence rate and different neuropsychological performance (46–48), while the G allele of the same polymorphism has been linked with symptoms of appetite disturbances in females (49). A correlation with violent suicide attempts was shown for other SNPs in *CLOCK* and *TIMELESS*, while the latter is also associated with the lifetime number of suicide attempts and a positive family history of suicide (50). A VNTR of *PER3* gene was shown to influence the general age of onset, as well as a postpartum depressive onset of the disorder (51, 52). *PER3* was also linked to an increased preference for the evening hours in daily activity among BD patients (42). Maciukiewicz et al. observed further associations between SNPs of *ARNTL* variants with sleep, appetite and depressive dimensions in BD (49).

A functional SNP in the promoter region of the *GSK3beta* gene *(nt* −*171 to* +*29*), which also shows a general association to impulsivity and suicide risk among patients with bipolar disease, was found to influence the age at onset of BD, as well as the response to treatment with antidepressant, lithium salts and chronotherapeutics (53–55). This polymorphism was recently shown to also influence white matter microstructure of bipolar patients under ongoing lithium treatment (56) and gray matter volumes in areas critical for the generation and control of affect implicated in BD pathophysiology (57).

Other polymorphisms influencing treatment response, such as the mood stabilizer effect of lithium salts (variant in the promoter of *NR1D1*) and a general association with positive treatment response (*CRY1)* have been described (58). Finally, Sjöholm et al. identified two risk haplotypes and one protective haplotype in the *CRY2* gene associated with rapid cycling in BD (59) (see Supplementary Table 1).

## Genetics of seasonality and seasonal affective disorder

The interplay between mood variations and seasonal rhythms in humans has received renewed interest since the diagnosis of Seasonal Affective Disorder (SAD) was proposed by Rosenthal in 1984, as “a condition characterized by recurrent depressive episodes that occur annually at the same time each year” [(60), p. 72]. The observation that many adults experience a “subsyndromal SAD”, with milder vegetative symptoms in the fall/winter months (61, 62), suggested that “seasonality may be a dimensional process rather than a discrete syndrome” [(63), p. 315].

### Serotonergic genes

Although the genetic basis of seasonality and SAD has not yet been completely identified, several studies suggest that both conditions have an inherited component (64–66). From a pathophysiological point of view, the typical symptoms of SAD, such as overeating, carbohydrate craving, weight gain, and oversleeping, point to a dysfunction of the serotonergic system (66). Moreover, the serotonin level in the human hypothalamus shows seasonal variations, with a general decrease during the winter season (67). The serotonin hypothesis is also supported by the large therapeutic evidence that selective serotonin reuptake inhibitors (SSRIs) and bright light therapy are effective in winter SAD (68–71), with reversion of this effect by rapid tryptophan depletion (70, 72).

Therefore, the first pioneer genetic studies focused on the molecular components of the serotonergic system (73). Rosenthal et al. showed that the short (s), as opposed to the long (l), allele of the 5-HT transporter linked polymorphism *(5-HTTLPR)* contributes to the trait of seasonality and is a risk factor for SAD (74). First reports showing an association of this variant with general susceptibility and several features of the clinical course among patients with SAD (75–77) could not be corroborated by a meta-analysis by Johansson et al., but the authors concluded that the polymorphism may have an effect on seasonal behavioral traits (78, 79).

Recent Positron Emission Tomography (PET) studies showed a significantly higher activity of serotonin transporter binding potential in several brain regions, during fall and winter, compared to spring and summer, in healthy volunteers (80, 81). Furthermore, “the first [11C]DASB PET longitudinal study investigating whole-brain seasonal 5-HTT fluctuations in both patients with SAD and in healthy individuals reported that a whole-brain seasonal change in 5-HTT predicted symptom severity in patients with SAD, an effect primarily driven by females with the short *5-HTTLPR* genotype (S' carriers)” [(82), p. 2], (83). These findings were later confirmed by other groups (83, 84).

The serotonin *5-HT2A* receptor gene has also been proposed as major candidate gene in association studies of seasonality and SAD (85, 86). In particular, it has been suggested that “downregulation of 5-HT2A receptors may underlie the therapeutic effects of SSRIs” [(64), p. 656], (87) and the effectiveness of light therapy in the treatment of SAD has also been linked to an alteration of the sensitivity of 5-HT2A receptors (76). Moreover, specific sequence polymorphisms in the coding region of the serotonin *5-HT2A* receptor gene have been found to be associated with the clinical features and course of depressive disorder or directly with seasonality and SAD (64, 86, 88–90).

### Circadian genes

Apart from an extensive connection between SAD and the serotoninergic system, genes of the core clock family have also been implicated in the disease. After a first report of a SNP in *NPAS2* being linked to SAD (91), Partonen et al. found further SNPs of *PER2, ARNTL*, and *NPAS2* to be associated with seasonality and SAD (92, 93).

Kim et al. also reported an association of *NPAS2* and *ARNTL*, especially with the metabolic components of seasonality (body weight and appetite). In addition, they found increased seasonal variations of mood and behavior among individuals carrying a *CLOCK* polymorphism previously implicated in bipolar disorder (40, 46–48, 94). These recent findings are in contrast with a previous work from the same group, showing that the same SNP of *CLOCK* is not associated with seasonal fluctuations in a sample of Korean college students (95).

Furthermore, another recent investigation highlighted the impact of two rare genetic variants of the *PERIOD3 gene (PER3)* on a circadian phenotype and a seasonal mood trait, which may be especially critical under conditions of short photoperiod (e.g., during the winter season) (19).

### Other genetic findings

Environmental light detection in humans is mediated by melanopsin containing intrinsically photosensitive retinal ganglion cells (ipRGCs), which are located in the inner retina (96–98). Some polymorphisms of the melanopsin gene may be linked to a greater sensitivity to light, thus determining functional variations in ipRGC activity. During shortened photoperiods (e.g., during the winter months) this may contribute to inter-individual differences in sleep and alertness (99, 100). A missense variant *(P10L)* in the melanopsin *(OPN4)* gene, which has also been found in SAD patients, has been proposed to contribute to changes in melanopsin sensitivity (99). Reduced retinal light sensitivity, especially during the winter months, as a pathophysiological hypothesis of SAD (101–103) recently gained first supporting evidence. A study by Roecklein et al. found a reduced post-illumination pupil response (PIPR) in SAD patients, compared with controls, in winter but not in summer (104).

A study by Delavest et al. investigating the *rs2072621* polymorphism of the X-linked *GPR50 gene*, a member of the G protein-coupled melatonin receptor subfamily, found an association with SAD in females, thus providing the first potential gender-specific molecular link between the hormone melatonin and SAD (105).

Yang et al. studied the relationship between *ST8SIA2* and *NCAM1*, two genes forming the polysialic acid neural cell adhesion molecule (NCAM) complex in the SCN, and circadian preferences, as well as seasonality, in healthy adult Korean subjects. The association of 8 SNPs of *ST8SIA2* and 2 SNPs of *NCAM1* with seasonality remained significant after correction for multiple testing (106).

Another study by Nam et al. found that the *GNB3* (G-protein β3 subunit) *C825T* polymorphism, which is associated with various medical conditions (107, 108) and psychiatric disorders, including recurrent winter depression or SAD (109, 110), also plays a role in seasonal variations in mood, body weight, energy level, and appetite, particularly in females.

## Conclusions

Gene polymorphisms of the core clock machinery and seasonal changes of the light-dark cycle substantially impact on the behavior of patients with mood disorders. The relationship between biological clock and behavior suggests a specific sensibility of these patients to psychobiological factors that can modify the circadian timing system, such as environmental synchronizers (light phase and seasonal photoperiod changes), and conditions directly perturbing the clock (sleep deprivation, or phase advance/delay). These factors can trigger or worsen the severity of mood disorders, but also be successfully exploited to treat manic and depressive episodes (111).

Current models of circadian homeostasis suggest that the hierarchical control exerted by the SCN on circadian rhythms of behavior, physiological functions, and on peripheral clocks (112), interacts with homeostatic mechanisms that also contribute to these phenomena. In rodents, a similar dependence of behavior on clock gene mutations occurs in the absence of other regulators of circadian rhythmicity, such as melatonin, and is abolished when these homeostatic components are restored (113). Therefore, we suggest that the high sensitivity of mood-disordered patients to clock gene variants is underpinned by a deficit in homeostatic mechanisms regulating the circadian timing system. Recent discoveries in humans of yet unknown circulating substances affecting the circadian phenotype and overcoming the timing of the clock gene machinery (114, 115), lead to hypothesize that a systematic investigation of these mechanisms will shed new light on the nature of circadian disruption in mood disorders.

## Author contributions

Both authors certify that they have participated sufficiently in the work to take public responsibility for the content, including participation in the concept, design, writing, or revision of the manuscript. In particular: CG conceived, designed and drafted the manuscript. FB drafted and critically reviewed the manuscript. CG and FB approved the final version of the manuscript.

### Conflict of interest statement

The authors declare that the research was conducted in the absence of any commercial or financial relationships that could be construed as a potential conflict of interest.
