# Supplementary material for: Genetic Factors Affecting Seasonality, Mood, and the Circadian Clock
Source: Front Endocrinol (Lausanne). 2018 Aug 23;9:481. doi: 10.3389/fendo.2018.00481 (PMC6115502; doi:10.3389/fendo.2018.00481)
Supplement: Supplementary file 1 [file Table_1.docx]

**Table 1:** Associations between single nucleotide polymorphisms (SNPs) of different circadian genes and mood disorders. BD: bipolar disorder, DD: depressive disorder, SAD: seasonal affective disorder.

| Gene | OMIM nomenclature | Polymorphism | Disease | Associated phenotype | Reference |
| --- | --- | --- | --- | --- | --- |
| *ARNTL* | ARYL HYDROCARBON RECEPTOR NUCLEAR TRANSLOCATOR-LIKE | rs7107287 | BD | General association | (36) |
|  |  | rs3789327  rs11022778  rs10766075 | BD | General association, sleep, appetite and depressive dimension of BD | (38,51) |
|  |  | rs2290035 | SAD | General association | (95) |
| *BHLHE40* | BASIC HELIX-LOOP-HELIX DOMAIN  CONTAINING, CLASS E, 40 | rs6442925 | BD | General association | (32,45) |
| *CLOCK* | CIRCADIAN LOCOMOTOR OUTPUT CYCLES KAPUT | rs1801260 | BD | General association, insomnia, higher evening activity and delayed sleep onset, higher episode recurrence rate | (42,48-50) |
|  |  | rs3805148  rs534654 | BD | Violent suicide attempts | (52) |
|  |  | rs17777927 | BD | General association | (38) |
| *CRY1* | CRYPTOCHROME 1 | rs2287161 | DD | General association | (29,30) |
|  |  | rs8192440 | BD | Positive response to lithium | (60) |
| *CRY2* | CRYPTOCHROME 2 | rs1554338 | BD | General association | (37) |
|  |  | rs10838524 | BD | Prone to rapid cycling | (61) |
|  |  | rs10838527  rs3824872 | BD | Protective of rapid cycling | (61) |
|  |  | rs4132063 | BD | General association | (32,45) |
| *CSNK1D* | CASEIN KINASE I ISOFORM DELTA | rs4510078 | BD | General association | (38) |
| *CSNK1E* | CASEIN KINASE 1 EPSILON | rs1534891 | BD | General association | (38) |
| *GSK3beta* | GLYCOGEN SYNTHASE KINASE 3 | rs334558 | BD | Association with BD2 in women | (43) |
|  | BETA | N.N. | BD | General association | (32,45) |
|  |  | promoter region (nt −171 to +29) | BD | Age at onset of the disorder,  response to treatment | (55-57) |

| Gene | OMIM nomenclature | Polymorphism | Disease | Associated phenotype | Reference |
| --- | --- | --- | --- | --- | --- |
| *NPAS2* | NEURONAL PAS DOMAIN PROTEIN 2 | rs1374324 | DD | General association, onset within 3 yrs | (27) |
|  |  | rs11123857  rs17025005 | DD | General association | (29,37) |
|  |  | rs11541353 | SAD | General association | (94,95) |
| *NR1D1* | NUCLEAR RECEPTOR SUBFAMILY 1,  GROUP D, MEMBER 1 | rs7502912 | DD | General association | (32) |
|  |  | rs2071427 | BD | Influence mood stabilizer effect of lithium | (60) |
|  |  | rs2314339 | BD | Increased preference to the evening hours in daily activities | (44) |
| *PER2* | PERIOD HOMOLOG 2 (DROSOPHILA) | rs10462023 | DD | General association, onset within 3 yrs | (27) |
|  |  | rs56013859 | SAD | General association | (95) |
| *PER3* | PERIOD HOMOLOG 3 (DROSOPHILA) | rs228697 | DD | Increased preference to the evening hours in daily activities | (44) |
|  |  | rs8192440 | BD | Positive response to lithium | (60) |
| *RORA* | RAR-RELATED ORPHAN RECEPTOR A | rs1554338 | BD | General association | (37) |
|  |  | rs782931 | BD | General association | (41) |
| *RORB* | RAR-RELATED ORPHAN RECEPTOR B | rs10491929 | BD | General association | (37) |
|  |  | rs7022435  rs3750420  rs1157358  rs3903529 | BD | Association with BD1 | (37) |
| *TIMELESS* | TIMELESS HOMOLOG (DROSOPHILA) | rs7486220 | DD | Excessive daytime fatigue among women | (8) |
|  |  | rs1082214  rs2291739 | DD | Early-morning awakening among men | (26) |
|  |  | rs2291738  rs2279665  rs774045 | BD | General association | (36,41) |
| *VIP* | VASOACTIVE INTESTINAL PEPTIDE | rs17083008 | BD | General association | (29) |
